# Identification of a Binding Site for ASF/SF2 on an RNA Fragment Derived from the Hepatitis *delta* Virus Genome

**DOI:** 10.1371/journal.pone.0054832

**Published:** 2013-01-18

**Authors:** Dorota Sikora, Dajiang Zhang, Teodora Bojic, Yasnee Beeharry, Ali Tanara, Martin Pelchat

**Affiliations:** Department of Biochemistry, Microbiology and Immunology, Faculty of Medicine, University of Ottawa, Ottawa, Canada; Saint Louis University, United States of America

## Abstract

The hepatitis *delta* virus (HDV) is a small (∼1700 nucleotides) RNA pathogen which encodes only one open reading frame. Consequently, HDV is dependent on host proteins to replicate its RNA genome. Recently, we reported that ASF/SF2 binds directly and specifically to an HDV-derived RNA fragment which has RNA polymerase II promoter activity. Here, we localized the binding site of ASF/SF2 on the HDV RNA fragment by performing binding experiments using purified recombinant ASF/SF2 combined with deletion analysis and site-directed mutagenesis. In addition, we investigated the requirement of ASF/SF2 for HDV RNA replication using RNAi-mediated knock-down of ASF/SF2 in 293 cells replicating HDV RNA. Overall, our results indicate that ASF/SF2 binds to a purine-rich region distant from both the previously published initiation site of HDV mRNA transcription and binding site of RNAP II, and suggest that this protein is not involved in HDV replication in the cellular system used.

## Introduction

**Figure 1:**
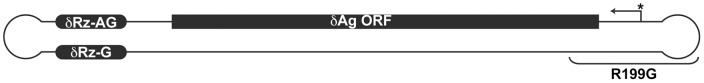
Representation of the hepatitis *delta* virus RNA genome. Both genomic (G) and antigenomic (AG) polarities of HDV RNA are superimposed; location of *delta* ribozyme (*delta* Rz) on each polarity is shown. The HDAg (delta Ag) coding region is indicated by the black rectangle and the transcription start site is depicted by an arrow and an asterisk. The location of the R199G fragment is indicated.

The hepatitis *delta* virus (HDV) consists of a small (∼1700 nucleotides), negative strand, circular RNA molecule folding into an unbranched, rod-like structure due to about 70% self-complementarity (Figure 1; for a review see [1]). The HDV RNA genome contains two complementary ribozyme motifs (i.e. *delta* Rz), and has a single open reading frame on antigenomic HDV RNA encoding two viral proteins (HDAg-S). These two proteins are mostly identical in sequence except that the large HDAg (HDAg-L) contains 19 additional amino acids at its C-terminus resulting from RNA editing of the antigenomic HDV RNA located on the termination codon of the small HDAg (HDAg-S) gene to a tryptophan codon [2]. HDAg-S is essential for HDV replication [3], [4], while the HDAg-L was reported to be required for virion assembly and to be an inhibitor of HDV replication [5]–[9].

HDV requires the hepatitis B virus (HBV) envelope proteins for dissemination [10]. However, HDV replication is entirely independent of the helper virus and uses host-encoded RNA polymerase II (RNAP II) to replicate its RNA genome without DNA intermediates [11]–[17]. HDV RNA replication takes place in the nucleus of infected cells, and is proposed to occur via a symmetrical double rolling circle mechanism [1]. According to this model, the circular HDV genome is used to produce linear antigenomic RNAs, which are either processed by polyadenylation to become HDAg mRNA, or are cleaved to unit-length RNAs by intrinsic *delta* Rz, and ligated to produce circular molecules. Likewise, transcription of these circular antigenomes produces linear genomic multimers, which are processed into unit-length circular genomes.

The right terminal stem-loop domain of genomic HDV RNA is believed to contain RNA promoter elements for RNAP II [11], [13], [18]. Transcription of the HDAg mRNA is proposed to be initiated at a unique site located in the top strand of the right terminal stem-loop domain of genomic HDV RNA (Figure 1, arrow and asterisk; [14]), although it is not known whether the initiation site of antigenomic RNA synthesis is identical to that of mRNA transcription. This region of HDV RNA acts as an RNA promoter *in vitro* [11], [18], and co-immunoprecipitates with RNAP II [13]. Mutations introduced in this region resulted in decreased HDV RNA accumulation in cell culture [18]–[20]. Notably, the rod-like conformation of this region was essential for RNAP II binding and transcription initiation *in vitro* [13], [18]. Recently, the formation of an active RNAP II pre-initiation complex on an RNA fragment corresponding to this region was demonstrated [11]. This complex closely resembled the one that forms on a canonical DNA promoter.

HDV has a very limited coding capacity and uses numerous host proteins to ensure its replication. Several screening approaches have found that both HDAg and HDV RNA interact with various host factors at different stages of the viral life cycle [21]. Although the biological significance of these findings remains unclear, it is apparent that extensive interactions of HDV with its host have a great impact on HDV biology and pathogenicity. Recently, we identified several candidate host proteins that might be involved in HDV replication, using an RNA fragment with promoter activity and corresponding to 199 nt from the right terminal stem-loop domain of genomic HDV RNA (i.e. R199G; Figure 1; [22]). This study led to the identification of ASF/SF2 as a protein binding directly to R199G. Specifically, R199G bound to purified recombinant ASF/SF2 by electrophoretic mobility shift assay, and co-immunoprecipitated with ASF/SF2 from HeLa nuclear extract [22]. Furthermore, both polarities of HDV RNA co-immunoprecipitated with ASF/SF2 from HeLa cells replicating the HDV genome [22]. Molecular excess of non-related RNA did not alter association, and addition of homologous RNA competitor greatly reduced the interaction. Additionally, the use of either urea, unrelated antibodies or the beads alone was used to show the specificity of the interaction during the immunoprecipitations [22]. All of these confirmed specificity of the interaction between ASF/SF2 and HDV RNA.

ASF/SF2 is a member of a large family of proteins known as the serine arginine-rich (SR) proteins, which play essential roles in constitutive and alternative splicing [23], [24]. Structurally, ASF/SF2 has one classical RNA recognition motif (RRM) in its N-terminal region, followed by an additional degenerate RRM; the two RRMs are separated by an arginine/glycine-rich (RGG) linker region. An SR domain is found in its C-terminus, and is involved in protein-protein interactions [25]. During alternative splicing, the activity of ASF/SF2 is modulated by the C-terminal domain (CTD) of the largest subunit of RNAP II [26], [27]. This interaction is involved in coupled transcription-splicing regulation, in which the CTD facilitates the assembly of early splicesome complexes [28]. In addition to splicing, ASF/SF2 plays important roles in several mRNA-processing pathways, such as mRNA export [29], [30], mRNA stability [31] and mRNA translation [32], and maintenance of genome integrity [33].

Because ASF/SF2 is a very abundant host protein, and due to its specific binding to both R199G and the CTD of RNAP II, it was hypothesized that ASF/SF2 could play a role in HDV replication [22]. Consequently, our objectives were to locate the binding site of ASF/SF2 on R199G, and to investigate the requirement of ASF/SF2 for HDV RNA accumulation. To locate the ASF/SF2 binding site on R199G, we performed binding experiments using purified recombinant ASF/SF2 combined with deletion analysis and site-directed mutagenesis of the RNA fragment. Using shRNAs targeting ASF/SF2 mRNA, we investigated the requirement of the protein for HDV RNA accumulation in 293 cells replicating the HDV RNA genome. Overall, our results indicate that ASF/SF2 binds to a purine-rich region downstream from the HDAg mRNA transcription initiation site of R199G, and suggest that this protein is not involved in HDV replication in the cellular system used.

## Materials and Methods

### Synthesis of RNA fragments

HDV RNA fragments, as well as the P11.60 RNA, were transcribed from DNA as previously described [22]. Biefly, the RNAs were synthesized from DNA templates generated by PCR amplification of sequences of pHDVd2, which is a derivative of pBluescriptKS+ (Stratagene) containing a dimeric HDV cDNA insert [22]. During PCR amplification, the forward primers were designed to contain a T7 promoter sequence. Unit-length genomic HDV RNA was synthesized from *Hin*DIII-linearized pHDVd2. RNAs were synthesized by *in vitro* transcription from DNA templates (1 μg), using 200 U of T7 RNA polymerase (New England Biolabs; NEB) in 1X transcription buffer (80 mM Tris-HCl, pH 7.9, 40 mM dithiothreitol, 20 mM MgCl_2_, 2 mM spermidine), with 5 mM of each NTP. Transcription reactions were carried out at 37°C for 4 h. DNA templates were removed by incubation with 10 U DNase I (Promega) at 37°C for 30 min. Products were resolved on either agarose gel or denaturing 5% polyacrylamide/7 M urea gel in 1X Tris-Borate-EDTA (TBE) buffer (90 mM Tris-HCl, pH 8.3, 90 mM boric acid, 2 mM EDTA). Following electrophoresis, RNA bands were visualized by either SYBR green staining or UV shadowing, excised, and eluted in 500 mM ammonium acetate, 0.1% SDS, 10 mM EDTA overnight at room temperature. The eluted RNAs were then ethanol-precipitated in the presence of 0.3 M NaOAc, pH 5.0, resuspended in H_2_O, passed through a Sephadex G-50 column (GE Healthcare), and ethanol-precipitated for a second time. RNA was quantified by spectrophotometry at a wavelength of 260 nm.

### His-ASF/SF2 expression and purification

C-terminally hexahistidine (his) –tagged ASF/SF2 was expressed from plasmid pET-20b(+)-ASF transformed into *Escherichia coli* strain BL21(DE3)pLysS (Novagen). Bacterial cultures were grown for 18 h at 37°C in Terrific Broth (Gibco) supplemented with 100 μg/μL of ampicillin, and cells were collected in 50-mL tubes following a 15-minute centrifugation at 6,000× g at 4°C. Pelleted cells were rotated in binding buffer (5 mM imidazole, 0.5 M NaCl, 20 mM Tris-HCl, pH 7.9, 8 M urea) for 60 min at room temperature, and cell lysate was cleared by centrifugation at 14,000× g for 30 min. His-tagged ASF/SF2 was purified by immobilized metal affinity chromatography using the Ni-NTA Spin columns (Qiagen) according to manufacturer's instructions. The column-bound proteins were washed 5 times with binding buffer containing 40 mM of imidazole, and eluted twice with elution buffer (500 mM imidazole, 0.5 M NaCl, 20 mM Tris-HCl, pH 7.9, 8 M urea). The protein extracts were dialyzed overnight at 4°C against 50 mM Tris-HCl, pH 7.9, 1 mM EDTA, 0.1 mM dithiothreitol, 50 mM NaCl, 50% glycerol, 1.5 M urea. Protein purity was evaluated by SDS-PAGE. To confirm the identity of the protein, following SDS-PAGE, the proteins were transferred to a nitrocellulose membrane (GenScript). Membrane was blocked in 5% non-fat dry milk in TBS buffer (50 mM Tris-HCl, pH 7.5, 1.25 M NaCl) +0.1% tween 20, and probed with a mouse monoclonal anti-ASF/SF2 antibody (Invitrogen). The membrane was then washed with TBS +0.1% tween 20, and incubated with a horse radish peroxidase-conjugated goat anti-mouse IgG antibody (Abcam). Protein was detected by incubation with the SuperSignal West Pico Chemiluminescent Substrate (Thermo Scientific), and visualized following exposure to X-ray film. Protein concentration was determined using the Bio-Rad Protein Assay Kit II (Bio-Rad), according to manufacturer's instructions. Protein aliquots were stored at −80°C.

### Fluorescence spectroscopy

Intrinsic fluorescence was measured with a Cary Eclipse fluorescence spectrophotometer (Agilent Technologies) using the Cary Eclipse Scan Application software. All binding experiments were carried out at room temperature, using an excitation wavelength of 280 nm. Emission spectra were read between 300–400 nm. Binding experiments were carried out in 100 mM KCl, 20 mM Tris-HCl, pH 7.5. Background emission was eliminated by subtracting the signal of buffer alone, or buffer plus His-ASF/SF2. Binding assays were carried out at an His-ASF/SF2 concentration of 100 nM. Changes in His-ASF/SF2 fluorescence upon addition of RNA ligand were fitted to a one site binding equation (i.e. –ΔF/F_o_  =  Bmax*[RNA]/(appK_D_+[RNA])) by non-linear regression using GraphPad Prism 3.0.

### Bioinformatics analysis

Sequences corresponding to the ASF/SF2 binding site from 92 HDV strains were obtained from the online Subviral RNA Database (http://subviral.med.uottawa.ca; [34]). Sequences were aligned with the ClustalW multiple alignment program using default parameters [35], and adjusted manually. A sequence logo was produced from the multiple alignment using the web-based WebLogo application (http://weblogo.berkeley.edu; [36]).

### ASF/SF2 knock-down

293-HDV cells are derived from the human embryonic kidney cell line 293TRex; it expresses a single copy of HDAg-S cDNA under the control of a tetracycline (TET)-inducible promoter, and contains a mutated HDV RNA genome that is unable to produce HDAg [37]. The cells were cultured in Dulbeccòs modified Eagle medium supplemented with 10% fetal bovine serum, 5 mg/mL blasticidin and 200 mg/mL hygromycin B. Transfections of 293-HDV cells were performed using Lipofectamine 2000 (Invitrogen) following manufacturer's instructions. A ratio of 5 μl lipofectamine: 4 μg plasmid DNA was used. Transfection efficiencies were estimated visually following co-transfection with a plasmid encoding the green fluorescent protein (pGFP), provided by Dr. E. G. Brown (University of Ottawa). ASF/SF2 knockdown was achieved by transfection of 293-HDV cells with four different shRNA constructs (MissionTM TRC shRNA Target Set NM_003016; sh1 = NM_003016.x-1539s1c1, sh2 = NM_003016.x-1508s1c1, sh3 = NM_003016.x-879s1c1, sh4 = NM_003016.x-829s1c1; Sigma). An empty vector (pKLO.1-puro; Sigma) and a non-target shRNA construct (shNT; SHC002; Sigma) were used as negative controls. Expression of HDAg-S was induced 12 h post-transfection with TET at 1 μg/ml. Cells were collected 12 h post-induction, and RNA and protein were extracted for further analysis. To assess knock-down levels, 30 μg of total cellular protein were resolved by SDS-PAGE, and detected by Western blotting using either a mouse monoclonal anti-ASF/SF2 antibody (Invitrogen) or a mouse monoclonal anti-ß-actin antibody (Abcam), and exposed to X-ray films, as above.

### RNA/protein extraction

Extraction of RNA and proteins from cultured cells was performed using the TRIzol Reagent (Invitrogen), following instructions given by the manufacturer. Total RNA concentration was estimated by spectrophotometry. RNA quality was verified by denaturing 1% agarose/2.2 M formaldehyde gel electrophoresis. RNA samples were used directly for cDNA synthesis, or stored in aliquots at −80°C. Protein concentration was determined using Bio-Rad Protein Assay Kit II (Bio-Rad), according to manufacturer's instructions, and the protein samples were stored in 1% SDS at −20°C.

### Quantitative RT-PCR analysis

One μg of RNA extracted from 293-HDV cells was transcribed into cDNA using the iScript Select cDNA Synthesis Kit (Bio-Rad) and random primers. The cDNA samples were subjected to qPCR with the following primers: R199G HDV forward, 5′-GGAATTCTAATACGACTCACTATAGGG-^1541^ACTGCTCGAGGATCTCTTCTCTCC^1564^-3′, R199G HDV reverse, 5′- ^60^ACATCCCCTCTCGGTGCTG^41^ -3′; β-actin forward, 5′- CCATGTTTGTGATGGGTGTGAACCA -3′, β-actin reverse, 5′- ACCAGTGGATGCAGGGATGATGTTC -3′. qPCR was performed by Chromo4 Real-Time Detector (Bio-Rad) using the iQ SYBR Green Supermix (Bio-Rad). The PCR reaction was set up as follows: initial denaturation at 95°C for 3 min, followed by 40 cycles of 30 s at 95°C, 20 s at 55°C and 30 s at 72°C. Following amplification, melting curve analysis was performed. The levels of HDV expression were quantified and normalized to β-actin expression using the 2-ΔΔC_T_ method [38]. Amplification efficiencies of the two primer pairs were tested by initial qPCR performed at different dilutions of the template, and were found to be within 10%. Three technical and three biological replicates were performed for each sample.

## Results

### Fluorescence properties of His-ASF/SF2

**Figure 2:**
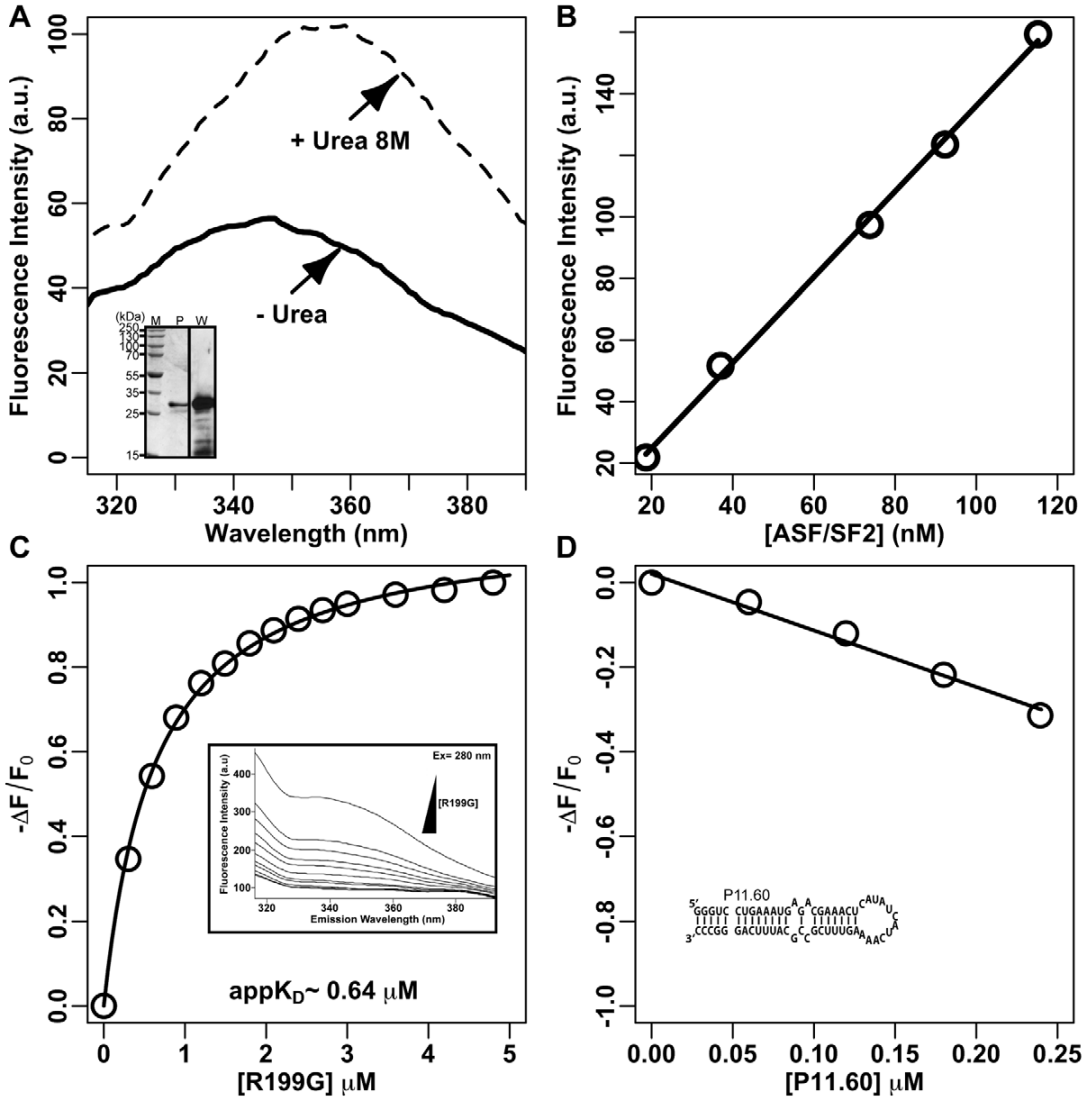
Fluorescence properties of His-ASF/SF2 and quantification of the binding of His-ASF/SF2 to R199G. (**A**) Emission spectrum of His-ASF/SF2 (100 nM) at an excitation wavelength of 280 nm. Solid line, purified protein in 20 mM Tris-HCl, 100 mM KCl, pH 7.5; Dashed line, purified protein after a 2 h incubation in 20 mM Tris-HCl, 100 mM KCl, pH 7.5, and 8 M urea; Inset, SDS-PAGE (P) and Western blot (W) of purified His-ASF/SF2. (**B**) Molar fluorescence of His-ASF/SF2. His-ASF/SF2 was excited at 280 nm and fluorescence intensity at increasing concentration of His-ASF/SF2 was measured at an emission wavelength of 340 nm. (**C**) Binding curve generated by plotting the observed change in fluorescence of His-ASF/SF2 as a function of increasing concentration of R199G. The curve was fitted to a one-site binding model using non-linear regression and the apparent K_D_ (appK_D_) was calculated. The inset represent typical ASF/SF2 emission spectra generated by the addition of increasing amount of R199G. (**D**) Variation of the observed change in fluorescence of His-ASF/SF2 as a function of increasing concentration of P11.60, a non-related RNA hairpin competitor. The insert represents the sequence and secondary structure of P11.60.

To investigate the binding of ASF/SF2 to R199G, we performed binding experiments with purified recombinant histidine-tagged ASF/SF2 (His-ASF/SF2) on R199G-derived RNAs using intrinsic tryptophan fluorescence spectrophotometry. To obtain a pure sample of the protein for this assay, His-ASF/SF2 was overexpressed in *E. coli* and purified under denaturing conditions followed by renaturation by stepwise dialysis, as previously described [28]. The purified protein was analyzed by SDS-PAGE, and the purity of the ∼33 kDa protein was estimated by densitometry to be over 90% (Figure 2A, lane P of the inset). Although the observed band migrated slightly slower than the expected calculated molecular weight of His-ASF/SF2 (i.e. 28.7 kDa), this protein was previously reported to typically migrate at 33 kDa [39], [40]. Furthermore, its identity was verified by Western blotting using a monoclonal anti-ASF/SF2 antibody (Figure 2A, lane W of the inset).

An emission spectrum of purified His-ASF/SF2 is shown in Figure 2A (solid line). The emission spectrum was obtained following excitation at 280 nm; this excitation wavelength was determined to produce the maximal emission peak after scanning the absorption spectrum of His-ASF/SF2. Emission maximum was observed at about 346 nm. Upon denaturation of the protein in 8 M urea, the emission maximum was red-shifted to 355 nm (Figure 2A; dashed line). This result is consistent with the exposure of the tryptophan residue to the polar solvent caused by the unfolding of the protein [41]. The fluorescence intensity also increased as a result of denaturation; increase in tryptophan fluorescence has been reported upon denaturation of proteins in urea [42], [43].

To verify that the intrinsic protein fluorescence is directly proportional to protein concentration, the molar fluorescence of His-ASF/SF2 was analyzed (Figure 2B). Fluorescence was measured at increasing concentrations of His-ASF/SF2, and was observed to be changing at a constant rate in the assayed concentration range (20–120 nM), indicating negligible loss of active protein through aggregation and/or adhesion. Based on these observations, all subsequent binding experiments were performed at an His-ASF/SF2 concentration of 100 nM. This concentration was both large enough to obtain a practicable fluorescence signal, and low enough to allow for an accurate estimation of apparent dissociation constants (appK_D_) between RNA fragments and His-ASF/SF2.

The interaction of His-ASF/SF2 and R199G RNA was studied by observing the response of tryptophan fluorescence to the addition of RNA. The binding experiments were carried out by adding increasing amounts of RNA to a fixed amount of His-ASF/SF2 (i.e. 100 nM), and observing a change in the emission spectra. The inset of Figure 2C shows typical emission spectra generated after the addition of RNA. The interaction of R199G RNA with His-ASF/SF2 caused a decrease in the fluorescence intensity but did not affect the position of the emission maximum, indicating that the fluorescence of tryptophan was quenched by the RNA. The quenching in tryptophan fluorescence following the addition of RNA indicated that the environment of the tryptophan residue was altered upon RNA binding. The binding curve produced from the observed change in the emission spectra of His-ASF/SF2 upon addition of increasing amounts of RNA is shown in Figure 2C. The difference in His-ASF/SF2 fluorescence was plotted as a function of increasing RNA concentration, and the results were fitted to a one-site binding curve using non-linear regression. An apparent dissociation constant (appK_D_) for the interaction, the concentration of the RNA at which 50% of the protein was bound, was calculated to be ∼0.64 μM under our experimental conditions. As a high appK_D_ is often associated with a low binding specificity, we tested His-ASF/SF2 binding to P11.60 RNA (a 60-nucleotide hairpin derived from the peach latent mosaic viroid; inset of Figure 2D); this RNA has been used as a non-related RNA hairpin competitor in previous studies and shown to not bind specifically to GST-ASF/SF2 [11], [13], [22], [44]. Binding of this RNA species increased the fluorescence of His-ASF/SF2 in a linear manner (i.e. giving negative –ΔF/F_0_), indicating that P11.60 binding to the protein is not specific and suggesting that P11.60 and R199G bind at distinct sites on the protein.

### Identification of an ASF/SF2 binding site on R199G RNA

**Figure 3:**
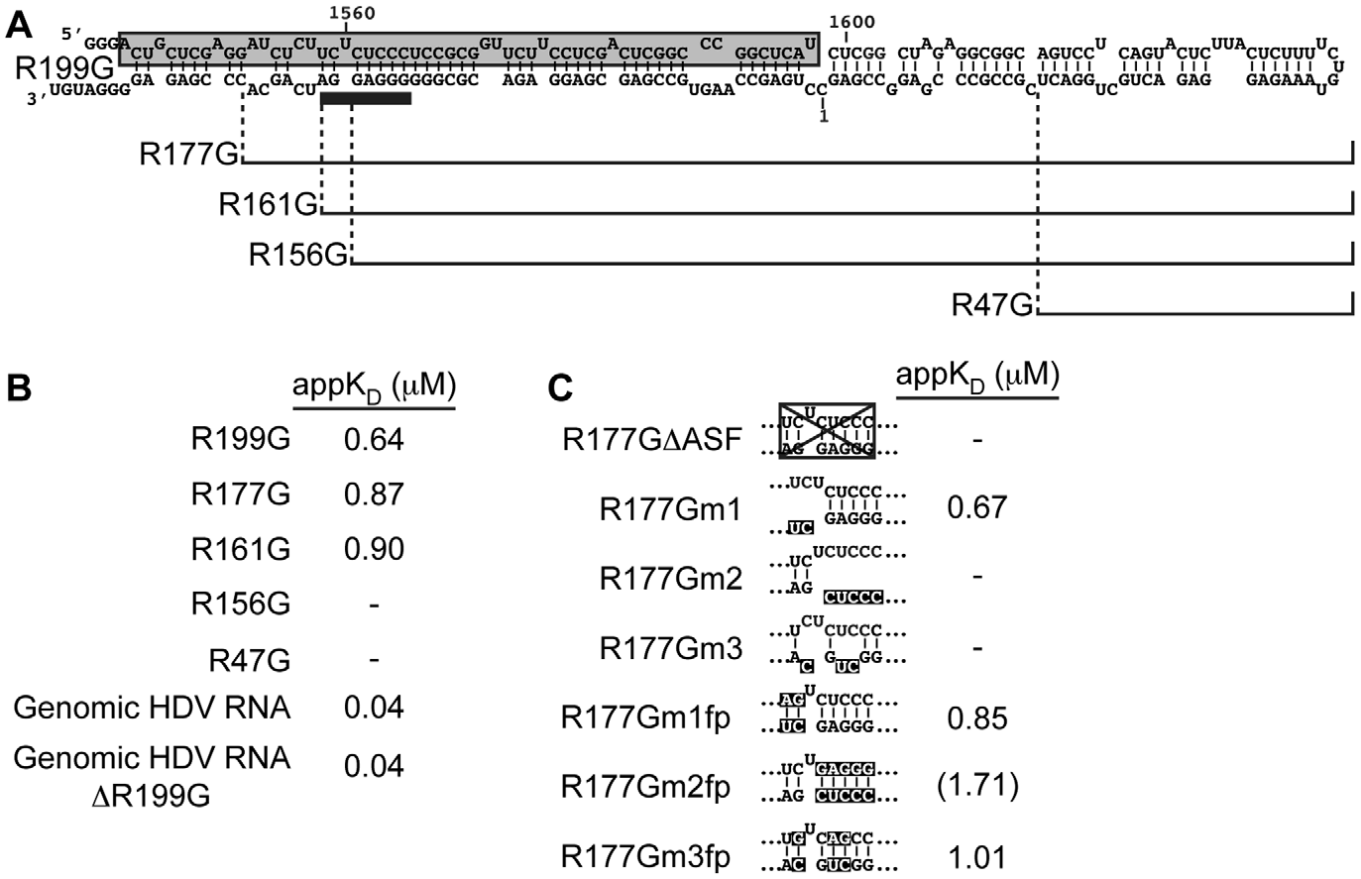
Identification of a binding site for His-ASF/SF2 on R199G RNA. (**A**) Representation of the RNA fragments used in comparison to R199G. The proposed His-ASF/SF2 binding site is indicated by the tick bar. (**B**) Binding affinities of His-ASF/SF2 to the R199G truncation mutants (R177G, R161G, R156G, and R47G), a unit-length HDV genomic RNA (Genomic HDV RNA) and a genomic HDV RNA without the R199G region (Genomic HDV RNA ΔR199G; positions 55 to 1540). (**C**) Binding affinities of His-ASF/SF2 to various R177G derivatives mutated at the proposed ASF/SF2 binding site. The box in R177ΔASF represents the deletion, and inverted fonts indicate mutations. As in Figure 2, excitation was performed at 280 nm, and emission was monitored at 340 nm. The curves were fitted using non-linear regression to a one-site binding model, and appK_D_(s) were calculated. Negative symbols indicate that the data could not be fitted to a one-site binding curve equation, consequently no appK_D_ could be calculated. Parentheses for the appK_D_ of R177Gm2fp indicate that this mutant baldy fits to a one-site binding curve equation and no saturation of the complex could be achieved.

Using this binding assay, we subsequently determined the location of the His-ASF/SF2 binding site on R199G. Truncation mutants were constructed and their interaction with His-ASF/SF2 was compared to that of R199G. Examination of the binding affinity of the R199G truncation mutants to His-ASF/SF2 revealed that shortening the stem of R199G to produce R177G only slightly increased the appK_D_ to ∼0.87 μM, suggesting that this deletion did not greatly affect the ability of the RNA to bind His-ASF/SF2 (Figure 3B). A similar affinity was observed between R161G RNA and His-ASF/SF2 (appK_D_ ∼0.90 μM). However, no appK_D_ could be calculated by reducing the stem further by testing either R156G or R47G. Based on these results, and previous findings that ASF/SF2 specifically binds polypurine stretches [45]–[47], the putative His-ASF/SF2 binding sequence was proposed to be 5′-GGGAGGA-3′ (indicated by the tick bar in Figure 3A).

Because the use of short RNA fragments could assume conformations that do not exist in the rod-like structure of genomic HDV RNA, we verify that His-ASF/SF2 was also binding to the entire genomic HDV RNA. The calculated appK_D_ (∼0.04 mM; Figure 3B; Genomic HDV RNA) was an order of magnitude lower than that observed for R199G. This might indicate a stronger affinity for the complete rod-like genomic RNA, but also that multiple binding sites might be present on the complete genome, which might give a lower appK_D_. To test whether there are additional His-ASF/SF2 binding sites present on the genomic HDV RNA, a genomic HDV RNA without the R199G region (Figure 3B; Genomic HDV RNA ΔR199G) was synthesized and tested for His-ASF/SF2 binding. Examination of the binding affinity of genomic HDV RNA without the R199G region to His-ASF/SF2 revealed a similar appK_D_ to the complete genome, and indicates that additional His-ASF/SF2 binding sites are present on genomic HDV RNA.

To verify that the proposed His-ASF/SF2 binding sequence identified on right-terminal domain of genomic HDV RNA is involved in ASF/SF2 binding, the R177G RNA fragment was modified by removing the 5′-GGGAGGA-3′ sequence, along with the region it hybridizes to when forming the stem structure, producing the R177GΔASF mutant (Figure 3C). No appK_D_ could be calculated for this mutant, indicating loss of binding. To further demonstrate that this region is involved in His-ASF/SF2 binding, three R177G derivatives mutated at the proposed ASF/SF2 binding site were synthesized. Mutations of the last two nucleotides of the proposed motif did not significantly affected the binding of His-ASF/SF2 (Figure 3C; R177Gm1). However, no appK_D_ could be calculated for mutations located at the beginning of the motif (Figure 3C, R177Gm2 and R177Gm3). Because these deletions could result in alternative RNA foldings, three additional R177G derivatives containing compensatory mutations, and predicted to adopt the same folding as R177G, were tested. His-ASF/SF2 bound to the “flip” mutants, but binding was affected by mutations of the base pairs downstream of the bulged U. Noteworthy, when the five base pairs of the motif downstream of this U were flipped (i.e. R177Gm2fp), a higher appK_D_ was calculated for this mutant. However, the observed change in the emission spectra of His-ASF/SF2 upon addition of increasing amounts of this mutant baldy fitted to a one-site binding curve equation and no saturation of the complex could be achieved, suggesting lost of specificity of the binding of His-ASF/SF1 to this mutants.

**Figure 4:**
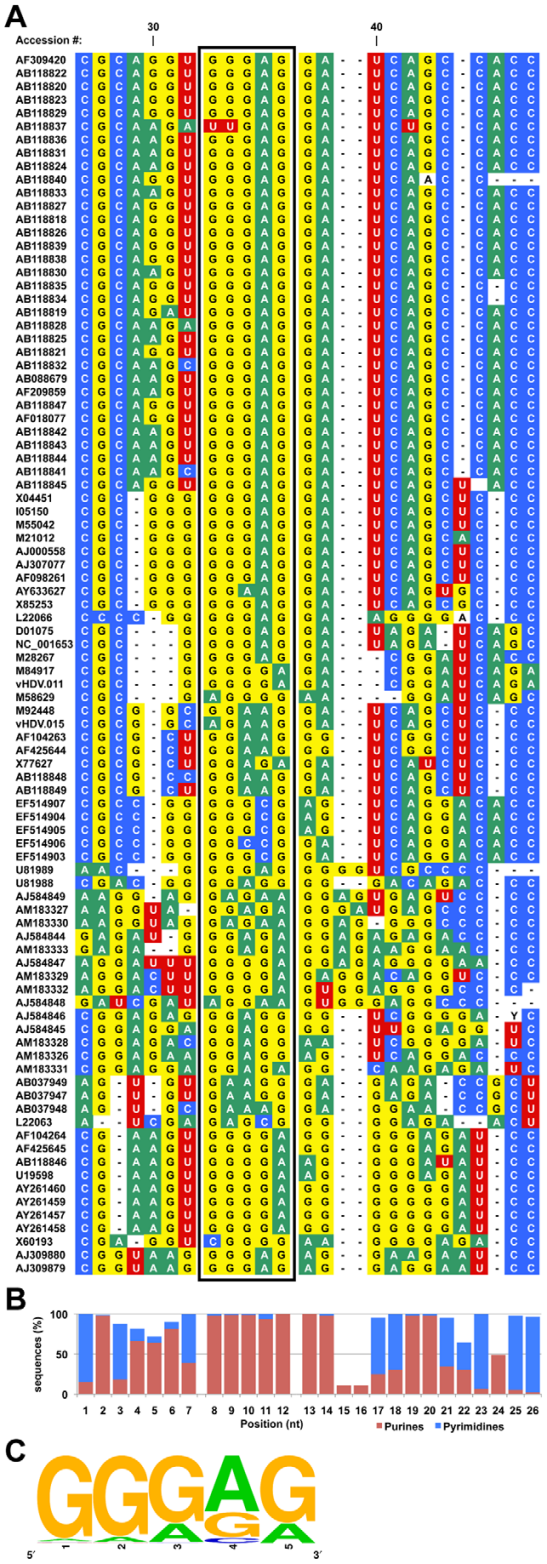
Conservation of the proposed ASF/SF2 binding site on HDV RNA. (**A**) Multiple alignment of sequences corresponding to the putative ASF/SF2 binding site in 92 HDV variants indexed in the Subviral RNA Database (http://subviral.med.uottawa.ca; [34]). (**B**) The percentage of purine and pyrimidine for each position was calculated. Blue and red indicate pyrimidine and purine, respectively. (**C**) Logo representation of the consensus sequence created from the sequence alignment of the putative ASF/SF2 binding site, which is indicated by a box in (A).

To investigate whether the proposed ASF/SF2 target sequence is conserved among different HDV strains, the region of HDV RNA containing the potential ASF/SF2 binding site was aligned across 92 variants of HDV (sequences obtained from the Subviral RNA Database; http://subviral.med.uottawa.ca; [34]; Figure 4A). The alignment revealed a conserved purine-rich sequence at the proposed ASF/SF2 binding site (Figure 4A, boxed nucleotides). The percentage of purine versus pyrimidine content at each nucleotide position was calculated (Figure 4B), and shows a strong enrichment in purine content at the proposed ASF/SF2 binding site across the aligned sequences. The obtained consensus sequence (Figure 4C) also strongly corresponds to the published ASF/SF2 recognition sequence (i.e. RGAAGARR; where R is purine; [47]), which was determined by a cross-linking immunoprecipitation and high-throughput sequencing (CLIP-seq) approach in cultured human embryonic kidney cells, suggesting that it is a genuine ASF/SF2 target.

### Effect of ASF/SF2 knock-down on the accumulation of HDV RNA in cell culture

The location of the binding site of ASF/SF2, distant from both the previously published initiation site of HDAg mRNA transcription and binding site of RNAP II [11], [13], [14], [18], suggests that this protein is not involved in RNA promoter recognition by RNAP II. To examine the potential involvement of ASF/SF2 in the replication of HDV, RNAi-mediated knock-down of ASF/SF2 was performed using four different plasmids containing shRNA constructs predicted to be directed against the 3′ untranslated region of ASF/SF2 mRNA. Under our condition, we observed cell death 48 hours after transfection with three of the four shRNA constructs in HeLa, 293, HepG2 and Huh7 cells. This is consistent with previous observations that the knock-down of ASF/SF2 induces G2 cell cycle arrest and apoptosis [48]. Because at earlier time points it might be difficult to differentiate HDV RNA accumulation from stability, we decided to use the HDV replication system developed by Chang et al. [37]. This replication system was created by stably transfecting a 293 cell line with a single copy of the HDAg-S cDNA under the control of a tetracycline (TET) -inducible promoter. These cells were then transfected with a mutated HDV RNA genome unable to produce HDAg-S, to create a cell line (designated 293-HDV) in which replication of HDV RNA is under the control of the TET-inducible HDAg-S. These cells are able to maintain a stable basal level of HDV replication in the absence of TET [37]. Upon tetracycline induction, these cells allows to measure the increase in HDV RNA accumulation at early time points, independently of the stability of HDV RNA present before induction.

**Figure 5:**
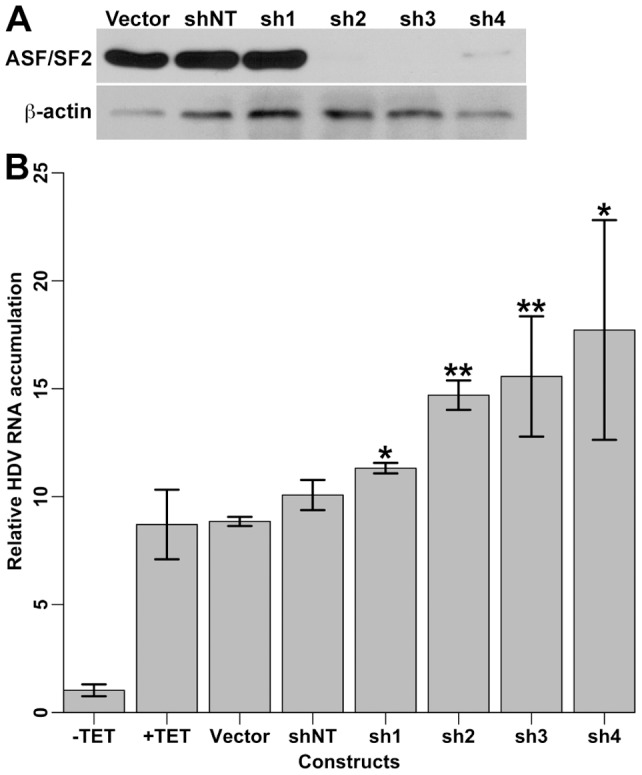
Effect of ASF/SF2 knock-down on HDV RNA accumulation. HDV-replicating 293 cells were transfected with plasmids harbouring different shRNAs against ASF/SF2 mRNA (sh1-4), a non-target shRNA (shNT), or an empty vector. (**A**) Representative of a Western blotting showing ASF/SF2 knock-down efficiency using ß-actin as a loading control. (**B**) HDV replication was induced by TET and HDV RNA accumulation was normalized to the amount of β-actin mRNA and quantified relative to non-induced cells by RT-qPCR analysis. A mean (+/− standard deviation) of three biological replicates is presented for each sample. Unpaired two-tailed t-test between HDV RNA accumulation from the sh1-4 treated cells and the shNT sample was performed: *, p-value <0.05; **, p-value <0.01.

Using the 293-HDV cell line as a model system for HDV replication, the cells were transfected with four different plasmids containing shRNA constructs directed against ASF/SF2 mRNA (sh1-4). Both a non-target shRNA construct (shNT) and the empty vector were used as negative controls. Transfection efficiency was monitored by co-transfection with a plasmid encoding the green fluorescent protein, and estimated visually to be at least 70–80%. The expression of HDAg-S, and thus induction of HDV RNA replication, was induced with TET (1 μg/μL) 12 hours post-transfection to minimize cell death. Cells were collected 12 hours later, and ASF/SF2 knock-down was verified by Western blotting (Figure 5A). When compared to the level of ß-actin, ASF/SF2 was not significantly reduced with one shRNA construct (sh1), while its level was very low to not detectable following transfection with the other three shRNAs where low cell survival was observed (sh2-4).

The effect of ASF/SF2 knock-down on the accumulation of HDV RNA was assessed by quantitative RT-PCR (qRT-PCR) using the level of β-actin mRNA to normalize the results. Upon TET induction, HDV RNA accumulation increased by ∼10-fold in non-transfected cells and in cells transfected with either a non-target shRNA construct or the empty vector (Figure 5B). Unexpectedly, the abundance of HDV RNA in anti-ASF/SF2 shRNA-transfected cells relative to non-target shRNA-transfected cells slightly increased following induction (Figure 5B). Also, these values inversely correlated with the amount of ASF/SF2 knockdown, where sh2-4 produced the largest effect. Although the increases in HDV accumulation is statistically significant, this effect is modest when compared to the two negative controls, and suggest that ASF/SF2 is not involved in HDV replication in this cellular system.

## Discussion

Previous experiments have identified ASF/SF2 as a binding partner of HDV RNA, both *in vitro* and in cells replicating the HDV RNA genome [22]. The HDV RNA fragment used as bait to identify the protein (i.e. R199G) includes the initiation site for HDAg mRNA transcription [14] and contains promoter elements to initiate transcription by RNAP II [11]. Moreover, mutagenesis of this segment affects HDV replication in several cellular systems [18]–[20]. Because ASF/SF2 interacts with the CTD of RNAP II [26], [27], it was hypothesized that ASF/SF2 could play a role in HDV replication at the step of RNA recognition by RNAP II [22]. Surprisingly, knock-down of ASF/SF2 in 293 cells replicating HDV resulted in less than two-fold increase in HDV RNA accumulation. ASF/SF2 plays important roles in various cellular processes [23], [24], [29]–[33], the slight increase in HDV RNA accumulation is likely due to indirect effects caused by the ASF/SF2 knock-down. As previously documented during ASF/SF2 knock-down [48], we observed cell death with the three shRNAs displaying very low to non-detectable levels of ASF/SF2. It is thus possible that in our cellular system, cell cycle arrest and/or apoptosis might have provided a better environment for HDV RNA accumulation, either directly by providing host factors for its accumulation, or indirectly by removing host factors involved in either the defence against HDV or its degradation. Because only a small change of HDV RNA accumulation was observed when ASF/SF2 was knock-downed, the implication of ASF/SF2 in HDV replication in this cellular system is likely not significant. However, since cells of non-hepatic origin was used, we cannot exclude the possibility that this protein might be implicated in HDV biology in hepatocytes or in the infected liver, where cell-type specific factors may exist. Also, the cellular system used in our study did not allow testing for cell-to-cell spread, interaction with the helper virus, virion assembly, or a link with either the production HDAgs or the activity of HDAg-L. Another untested possibility is that ASF/SF2 might have a role in HDV RNA cellular localization, since this protein has been shown to enhance cytoplasmic accumulation of an intronless mRNA [29].

To localize the binding site of ASF/SF2 on R199G, we performed binding experiments using purified recombinant His-ASF/SF2 combined with deletion analysis and site-directed mutagenesis on the RNA fragment. The fluorescence binding assay used to characterize the His-ASF/SF2-HDV RNA interaction is a technique frequently used to investigate protein-ligand interactions [49]–[52]. It provides an easy method of calculating an appK_D_ for the interaction by adding increasing concentrations of RNA to a fixed amount of protein and observing the resulting quenching of the intrinsic tryptophan fluorescence of the protein. Emission maxima of native proteins depend on the position of tryptophan residues in the folded protein. Typically, surface residues that are exposed to a polar solvent have an emission wavelength close to that of free tryptophan (i.e. emission maximum  = 348 nm for L-Trp in H_2_O; [53]), whereas residues that are buried in the interior of the protein will be blue-shifted (i.e. smaller emission maximum; [41]). ASF/SF2 has a single tryptophan residue located in one of its two RRMs. The observed emission maximum of His-ASF/SF2 at 346 nm indicates that the tryptophan is partially exposed to the solvent, in agreement with the solution structure of the RMM2 of ASF/SF2 in which the tryptophan residue is surface-exposed [54]. This tryptophan was previously demonstrated to be directly involved in binding to a short purine-rich RNA oligonucleotide [54]. Aromatic residues have been shown to be involved in single stranded nucleic acid binding in a number of proteins, either by polar interactions or by planar stacking interactions of the aromatic side chain with exposed bases [51], [55]–[60]. Hence, it is likely that the tryptophan of ASF/SF2 is also directly involved in binding to R199G RNA, although it is also possible that the RNA is binding somewhere else on the protein and affects its conformation, resulting in quenching of tryptophan fluorescence by other residues.

After testing a number of truncation mutants, the His-ASF/SF2 binding site was localized to a short purine-rich region. Sequence-specific binding of ASF/SF2 to short, purine-rich regions of RNA has been demonstrated, both *in vitro* [54], [61], [62] and *in vivo* [47], [63]. The identified ASF/SF2 binding site on HDV RNA highly resembles the ASF/SF2 target motif derived from many types of functionally distinct transcripts, including mRNAs, microRNAs, small nucleolar RNAs and non-coding RNAs [47], supporting the claim that the identified ASF/SF2 recognition sequence on R199G represents a physiological target of ASF/SF2. Curiously, the R199G RNA fragment contains multiple purine-rich regions that could theoretically serve as ASF/SF2 recognition sites. Sequence specific recognition of single-stranded RNA by several RRMs has been shown to be strongly reliant on the structural context of the target sequence (reviewed in [64]). It has been suggested that the interaction of the protein with the RNA structure induces conformational changes that allow for the sequence-specific RNA-RRM binding to occur [65], [66]. It is therefore possible that the identified purine-rich sequence on R199G is recognized by ASF/SF2 because it is found in a structurally optimal context. More importantly, the identification of the ASF/SF2 binding site away from the proposed initiation site for HDAg mRNA transcription [11], [14], combined with our observation of the effect of ASF/SF2 knock-down in cells replicating HDV RNA, indicate that ASF/SF2 is not directly involved in promoter recognition and/or transcription initiation by RNAP II. This conclusion is further supported by previous findings showing that only the tips of the rod-like structure of HDV RNA are required for both RNAP II binding and formation of a transcription pre-initiation complex [11], [13].

## Conclusions

In summary, we have shown that ASF/SF2 is binding R199G at a distinct conserved location downstream of the proposed transcription initiation site and RNAP II binding region. In addition, our results indicate that additional ASF/SF2 binding sites exist on the HDV RNA genome. Although ASF/SF2 appeared to be dispensable for HDV accumulation in the cellular system used in our study, its direct and specific association with HDV RNA suggests that it is possible that it plays a role in HDV biology. Also, although not investigated, post-translational modifications on ASF/SF2, such as methylation and phosphorylation, have been reported to change its activity in cells [67]–[69]. Further examination of the effect of those modifications in the context of viral replication might provide important insights into the role of this protein in the life cycle of HDV.

## References

[1] Taylor JM (2009) Chapter 3. Replication of the hepatitis delta virus RNA genome. Adv Virus Res 74: 103–121. 19698896 10.1016/S0065-3527(09)74003-5

[2] Casey JL (2012) Control of ADAR1 editing of hepatitis delta virus RNAs. Curr Top Microbiol Immunol 353: 123–143. 21732238 10.1007/82_2011_146 PMC3572862

[3] Kuo MY, Chao M, Taylor J (1989) Initiation of replication of the human hepatitis delta virus genome from cloned DNA: role of delta antigen. J Virol 63: 1945–1950. 2649689 10.1128/jvi.63.5.1945-1950.1989 PMC250607

[4] Yamaguchi Y, Filipovska J, Yano K, Furuya A, Inukai N, et al (2001) Stimulation of RNA polymerase II elongation by hepatitis delta antigen. Science 293: 124–127. 11387440 10.1126/science.1057925

[5] Chao M, Hsieh SY, Taylor J (1990) Role of two forms of hepatitis delta virus antigen: evidence for a mechanism of self-limiting genome replication. J Virol 64: 5066–5069. 2398535 10.1128/jvi.64.10.5066-5069.1990 PMC247998

[6] Chang FL, Chen PJ, Tu SJ, Wang CJ, Chen DS (1991) The large form of hepatitis delta antigen is crucial for assembly of hepatitis delta virus. Proc Natl Acad Sci USA 88: 8490–8494. 1924308 10.1073/pnas.88.19.8490 PMC52534

[7] Ryu WS, Bayer M, Taylor J (1992) Assembly of hepatitis delta virus particles. J Virol 66: 2310–2315. 1548764 10.1128/jvi.66.4.2310-2315.1992 PMC289026

[8] Sureau C, Moriarty AM, Thornton GB, Lanford RE (1992) Production of infectious hepatitis delta virus in vitro and neutralization with antibodies directed against hepatitis B virus pre-S antigens. J Virol 66: 1241–1245. 1309901 10.1128/jvi.66.2.1241-1245.1992 PMC240836

[9] Lee CZ, Chen PJ, Chen DS (1995) Large hepatitis delta antigen in packaging and replication inhibition: role of the carboxyl-terminal 19 amino acids and amino-terminal sequences. J Virol 69: 5332–5336. 7636976 10.1128/jvi.69.9.5332-5336.1995 PMC189372

[10] Rizzetto M, Canese MG, Arico S, Crivelli O, Trepo C, et al (1977) Immunofluorescence detection of new antigen-antibody system (delta/anti-delta) associated to hepatitis B virus in liver and in serum of HBsAg carriers. Gut 18: 997–1003. 75123 10.1136/gut.18.12.997 PMC1411847

[11] Abrahem A, Pelchat M (2008) Formation of an RNA polymerase II preinitiation complex on an RNA promoter derived from the hepatitis delta virus RNA genome. Nucleic Acids Res 36: 5201–5211. 18682525 10.1093/nar/gkn501 PMC2532721

[12] Filipovska J, Konarska MM (2000) Specific HDV RNA-templated transcription by pol II in vitro. RNA 6: 41–54. 10668797 10.1017/s1355838200991167 PMC1369892

[13] Greco-Stewart VS, Miron P, Abrahem A, Pelchat M (2007) The human RNA polymerase II interacts with the terminal stem-loop regions of the hepatitis *delta* virus RNA genome. Virology 357: 68–78. 16959288 10.1016/j.virol.2006.08.010

[14] Gudima S, Wu SY, Chiang CM, Moraleda G, Taylor J (2000) Origin of hepatitis delta virus mRNA. J Virol 74: 7204–7210. 10906174 10.1128/jvi.74.16.7204-7210.2000 PMC112241

[15] Moraleda G, Taylor J (2001) Host RNA polymerase requirements for transcription of the human hepatitis delta virus genome. J Virol 75: 10161–10169. 11581384 10.1128/JVI.75.21.10161-10169.2001 PMC114590

[16] Fu TB, Taylor J (1993) The RNAs of hepatitis *delta* virus are copied by RNA polymerase II in nuclear homogenates. J Virol 67: 6965–6972. 8230419 10.1128/jvi.67.12.6965-6972.1993 PMC238155

[17] Chang J, Nie X, Chang HE, Han Z, Taylor J (2008) Transcription of hepatitis delta virus RNA by RNA polymerase II. J Virol 82: 1118–1127. 18032511 10.1128/JVI.01758-07 PMC2224410

[18] Beard MR, MacNaughton TB, Gowans EJ (1996) Identification and characterization of a hepatitis delta virus RNA transcriptional promoter. J Virol 70: 4986–4995. 8764005 10.1128/jvi.70.8.4986-4995.1996 PMC190452

[19] Gudima S, Dingle K, Wu TT, Moraleda G, Taylor J (1999) Characterization of the 5′ ends for polyadenylated RNAs synthesized during the replication of hepatitis *delta* virus. J Virol 73: 6533–6539. 10400749 10.1128/jvi.73.8.6533-6539.1999 PMC112736

[20] Wu TT, Netter HJ, Lazinski DW, Taylor JM (1997) Effects of nucleotide changes on the ability of hepatitis delta virus to transcribe, process, and accumulate unit-length, circular RNA. J Virol 71: 5408–5414. 9188612 10.1128/jvi.71.7.5408-5414.1997 PMC191780

[21] Greco-Stewart V, Pelchat M (2010) Interaction of host cellular proteins with components of the hepatitis delta virus. Viruses 2: 189–212. 21994607 10.3390/v2010189 PMC3185554

[22] Sikora D, Greco-Stewart VS, Miron P, Pelchat M (2009) The hepatitis delta virus RNA genome interacts with eEF1A1, p54(nrb), hnRNP-L, GAPDH and ASF/SF2. Virology 390: 71–78. 19464723 10.1016/j.virol.2009.04.022

[23] Zahler AM, Lane WS, Stolk JA, Roth MB (1992) SR proteins: a conserved family of pre-mRNA splicing factors. Genes Dev 6: 837–847. 1577277 10.1101/gad.6.5.837

[24] Zahler AM, Neugebauer KM, Lane WS, Roth MB (1993) Distinct functions of SR proteins in alternative pre-mRNA splicing. Science 260: 219–222. 8385799 10.1126/science.8385799

[25] Birney E, Kumar S, Krainer AR (1993) Analysis of the RNA-recognition motif and RS and RGG domains: conservation in metazoan pre-mRNA splicing factors. Nucleic Acids Res 21: 5803–5816. 8290338 10.1093/nar/21.25.5803 PMC310458

[26] Cramer P, Caceres JF, Cazalla D, Kadener S, Muro AF, et al (1999) Coupling of transcription with alternative splicing: RNA pol II promoters modulate SF2/ASF and 9G8 effects on an exonic splicing enhancer. Mol Cell 4: 251–258. 10488340 10.1016/s1097-2765(00)80372-x

[27] McCracken S, Fong N, Yankulov K, Ballantyne S, Pan G, et al (1997) The C-terminal domain of RNA polymerase II couples mRNA processing to transcription. Nature 385: 357–361. 9002523 10.1038/385357a0

[28] Millhouse S, Manley JL (2005) The C-terminal domain of RNA polymerase II functions as a phosphorylation-dependent splicing activator in a heterologous protein. Mol Cell Biol 25: 533–544. 15632056 10.1128/MCB.25.2.533-544.2005 PMC543425

[29] Huang Y, Steitz JA (2005) SRprises along a messenger's journey. Mol Cell 17: 613–615. 15749011 10.1016/j.molcel.2005.02.020

[30] Caceres JF, Screaton GR, Krainer AR (1998) A specific subset of SR proteins shuttles continuously between the nucleus and the cytoplasm. Genes Dev 12: 55–66. 9420331 10.1101/gad.12.1.55 PMC316398

[31] Lemaire R, Prasad J, Kashima T, Gustafson J, Manley JL, et al (2002) Stability of a PKCI-1-related mRNA is controlled by the splicing factor ASF/SF2: a novel function for SR proteins. Genes Dev 16: 594–607. 11877379 10.1101/gad.939502 PMC155348

[32] Sanford JR, Gray NK, Beckmann K, Caceres JF (2004) A novel role for shuttling SR proteins in mRNA translation. Genes Dev 18: 755–768. 15082528 10.1101/gad.286404 PMC387416

[33] Li X, Manley JL (2005) Inactivation of the SR protein splicing factor ASF/SF2 results in genomic instability. Cell 122: 365–378. 16096057 10.1016/j.cell.2005.06.008

[34] Rocheleau L, Pelchat M (2006) The Subviral RNA Database: a toolbox for viroids, the hepatitis delta virus and satellite RNAs research. BMC Microbiol 6: 24. 16519798 10.1186/1471-2180-6-24 PMC1413538

[35] Thompson JD, Higgins DG, Gibson TJ (1994) CLUSTAL W: improving the sensitivity of progressive multiple sequence alignment through sequence weighting, position-specific gap penalties and weight matrix choice. Nucleic Acids Res 22: 4673–4680. 7984417 10.1093/nar/22.22.4673 PMC308517

[36] Crooks GE, Hon G, Chandonia JM, Brenner SE (2004) WebLogo: a sequence logo generator. Genome Res 14: 1188–1190. 15173120 10.1101/gr.849004 PMC419797

[37] Chang J, Gudima SO, Tarn C, Nie X, Taylor JM (2005) Development of a novel system to study hepatitis delta virus genome replication. J Virol 79: 8182–8188. 15956563 10.1128/JVI.79.13.8182-8188.2005 PMC1143748

[38] Livak KJ, Schmittgen TD (2001) Analysis of relative gene expression data using real-time quantitative PCR and the 2(-Delta Delta C(T)) Method. Methods 25: 402–408. 11846609 10.1006/meth.2001.1262

[39] Ge H, Zuo P, Manley JL (1991) Primary structure of the human splicing factor ASF reveals similarities with Drosophila regulators. Cell 66: 373–382. 1855257 10.1016/0092-8674(91)90626-a

[40] Krainer AR, Mayeda A, Kozak D, Binns G (1991) Functional expression of cloned human splicing factor SF2: homology to RNA-binding proteins, U1 70K, and Drosophila splicing regulators. Cell 66: 383–394. 1830244 10.1016/0092-8674(91)90627-b

[41] Eftink MR, Ghiron CA (1976) Exposure of tryptophanyl residues in proteins. Quantitative determination by fluorescence quenching studies. Biochemistry 15: 672–680. 1252418 10.1021/bi00648a035

[42] Benzaghou I, Bougie I, Picard-Jean F, Bisaillon M (2006) Energetics of RNA binding by the West Nile virus RNA triphosphatase. FEBS Lett 580: 867–877. 16413541 10.1016/j.febslet.2006.01.006

[43] Souliere MF, Perreault JP, Bisaillon M (2008) Magnesium-binding studies reveal fundamental differences between closely related RNA triphosphatases. Nucleic Acids Res 36: 451–461. 18039706 10.1093/nar/gkm1067 PMC2241848

[44] Greco-Stewart VS, Schissel E, Pelchat M (2009) The hepatitis delta virus RNA genome interacts with the human RNA polymerases I and III. Virology 386: 12–15. 19246067 10.1016/j.virol.2009.02.007

[45] Graveley BR (2000) Sorting out the complexity of SR protein functions. RNA 6: 1197–1211. 10999598 10.1017/s1355838200000960 PMC1369994

[46] Wang X, Juan L, Lv J, Wang K, Sanford JR, et al (2011) Predicting sequence and structural specificities of RNA binding regions recognized by splicing factor SRSF1. BMC Genomics 12 Suppl 5S8. 10.1186/1471-2164-12-S5-S8 PMC3287504 22369183

[47] Sanford JR, Wang X, Mort M, Vanduyn N, Cooper DN, et al (2009) Splicing factor SFRS1 recognizes a functionally diverse landscape of RNA transcripts. Genome Res 19: 381–394. 19116412 10.1101/gr.082503.108 PMC2661799

[48] Li X, Wang J, Manley JL (2005) Loss of splicing factor ASF/SF2 induces G2 cell cycle arrest and apoptosis, but inhibits internucleosomal DNA fragmentation. Genes Dev 19: 2705–2714. 16260492 10.1101/gad.1359305 PMC1283963

[49] Bougie I, Bisaillon M (2007) Characterization of the RNA binding energetics of the Candida albicans poly(A) polymerase. Yeast 24: 431–446. 17410550 10.1002/yea.1482

[50] Bougie I, Parent A, Bisaillon M (2004) Thermodynamics of ligand binding by the yeast mRNA-capping enzyme reveals different modes of binding. Biochem J 384: 411–420. 15307816 10.1042/BJ20041112 PMC1134125

[51] Elton D, Medcalf L, Bishop K, Harrison D, Digard P (1999) Identification of amino acid residues of influenza virus nucleoprotein essential for RNA binding. J Virol 73: 7357–7367. 10438825 10.1128/jvi.73.9.7357-7367.1999 PMC104262

[52] Flowers S, Biswas EE, Biswas SB (2003) Conformational dynamics of DnaB helicase upon DNA and nucleotide binding: analysis by intrinsic tryptophan fluorescence quenching. Biochemistry 42: 1910–1921. 12590577 10.1021/bi025992v

[53] Teale FW, Weber G (1957) Ultraviolet fluorescence of the aromatic amino acids. Biochem J 65: 476–482. 13412650 10.1042/bj0650476 PMC1199900

[54] Tintaru AM, Hautbergue GM, Hounslow AM, Hung ML, Lian LY, et al (2007) Structural and functional analysis of RNA and TAP binding to SF2/ASF. EMBO Rep 8: 756–762. 17668007 10.1038/sj.embor.7401031 PMC1978082

[55] Brun F, Toulme JJ, Helene C (1975) Interactions of aromatic residues of proteins with nucleic acids. Fluorescence studies of the binding of oligopeptides containing tryptophan and tyrosine residues to polynucleotides. Biochemistry 14: 558–563. 234245 10.1021/bi00674a015

[56] Gorlach M, Wittekind M, Beckman RA, Mueller L, Dreyfuss G (1992) Interaction of the RNA-binding domain of the hnRNP C proteins with RNA. EMBO J 11: 3289–3295. 1380452 10.1002/j.1460-2075.1992.tb05407.x PMC556863

[57] Jessen TH, Oubridge C, Teo CH, Pritchard C, Nagai K (1991) Identification of molecular contacts between the U1 A small nuclear ribonucleoprotein and U1 RNA. EMBO J 10: 3447–3456. 1833186 10.1002/j.1460-2075.1991.tb04909.x PMC453073

[58] Petrik J, Parker H, Alexander GJ (1999) Human hepatic glyceraldehyde-3-phosphate dehydrogenase binds to the poly(U) tract of the 3′ non-coding region of hepatitis C virus genomic RNA. J Gen Virol 80 (Pt 12): 3109–3113. 10.1099/0022-1317-80-12-3109 10567641

[59] Oubridge C, Ito N, Evans PR, Teo CH, Nagai K (1994) Crystal structure at 1.92 A resolution of the RNA-binding domain of the U1A spliceosomal protein complexed with an RNA hairpin. Nature 372: 432–438. 7984237 10.1038/372432a0

[60] Ueda H, Iyo H, Doi M, Inoue M, Ishida T (1991) Cooperative stacking and hydrogen bond pairing interactions of fragment peptide in cap binding protein with mRNA cap structure. Biochim Biophys Acta 1075: 181–186. 1932073 10.1016/0304-4165(91)90249-g

[61] Manley JL, Tacke R (1996) SR proteins and splicing control. Genes Dev 10: 1569–1579. 8682289 10.1101/gad.10.13.1569

[62] Smith PJ, Zhang C, Wang J, Chew SL, Zhang MQ, et al (2006) An increased specificity score matrix for the prediction of SF2/ASF-specific exonic splicing enhancers. Hum Mol Genet 15: 2490–2508. 16825284 10.1093/hmg/ddl171

[63] Sanford JR, Coutinho P, Hackett JA, Wang X, Ranahan W, et al (2008) Identification of nuclear and cytoplasmic mRNA targets for the shuttling protein SF2/ASF. PLoS ONE 3: e3369. 18841201 10.1371/journal.pone.0003369 PMC2556390

[64] Maris C, Dominguez C, Allain FH (2005) The RNA recognition motif, a plastic RNA-binding platform to regulate post-transcriptional gene expression. FEBS J 272: 2118–2131. 15853797 10.1111/j.1742-4658.2005.04653.x

[65] Allain FH, Gubser CC, Howe PW, Nagai K, Neuhaus D, et al (1996) Specificity of ribonucleoprotein interaction determined by RNA folding during complex formulation. Nature 380: 646–650. 8602269 10.1038/380646a0

[66] Showalter SA, Hall KB (2004) Altering the RNA-binding mode of the U1A RBD1 protein. J Mol Biol 335: 465–480. 14672656 10.1016/j.jmb.2003.10.055

[67] Sinha R, Allemand E, Zhang Z, Karni R, Myers MP, et al (2010) Arginine methylation controls the subcellular localization and functions of the oncoprotein splicing factor SF2/ASF. Mol Cell Biol 30: 2762–2774. 20308322 10.1128/MCB.01270-09 PMC2876523

[68] Misteli T (1999) RNA splicing: What has phosphorylation got to do with it? Curr Biol 9: R198–200. 10209090 10.1016/s0960-9822(99)80128-6

[69] Ong SE, Mittler G, Mann M (2004) Identifying and quantifying in vivo methylation sites by heavy methyl SILAC. Nat Methods 1: 119–126. 15782174 10.1038/nmeth715

